# Lifestyle and BMI Changes after the Release of COVID-19 Restrictions: Do Humans Go ‘Back to Normal’?

**DOI:** 10.3390/biology13110858

**Published:** 2024-10-23

**Authors:** Boris Krznar, Marijan Vilenica, Frank Rühli, Nicole Bender

**Affiliations:** Institute of Evolutionary Medicine, University of Zurich, Winterthurerstrasse 190, 8057 Zurich, Switzerland; boris.krznar@uzh.ch (B.K.); marijan.vilenica@gmail.com (M.V.); frank.ruehli@iem.uzh.ch (F.R.)

**Keywords:** overweight, pandemic, life history strategies, adaptation, diet, physical activity

## Abstract

Humans adapt their life history strategy and lifestyle behavior to the environment. The social restrictions during the COVID-19 pandemic in the years 2020–2021 led to changes in diet and physical activity habits in large parts of populations worldwide. Additionally, many people reported an increase in body weight during the pandemic. We investigated how diet quality, physical activity, and BMI changed since the end of the COVID-19 restrictions in Switzerland (2019–2023). We assessed diet and physical activity using a standardized questionnaire. We measured anthropometry and BMI using a stadiometer and a balance of a medical bioimpedance analyzer. Our results show a stable BMI in men and women during the pandemic. In both sexes, diet quality and physical activity levels did not change significantly during this time. Among women, physical activity and age were associated with BMI, while in men, apart from physical activity, high diet quality and living in the agglomeration were associated with BMI. Overall, we did not find evidence for a transient “unhealthier” lifestyle immediately after the COVID-19 pandemic. However, we found constituent sex differences in BMI determinants. Our results could indicate a higher resilience in highly educated persons and might contribute to more personalized approaches to prevent obesity.

## Simple Summary

Humans exhibit varied responses to natural disasters, including pandemics. Some increase their health behavior, while others decrease it. These differences are studied within the framework of the life history theory, where reactions to environmental challenges depend on age, sex, and socioeconomic status. We investigated diet, physical activity, and BMI in a Swiss population during and after the COVID-19 pandemic. We found no changes in health behaviors or BMI at this time. However, we found constant sex differences in several outcomes. While physical activity was associated with a lower BMI in both sexes, women showed an increase in BMI with increasing age, while men showed a lower BMI with a healthier diet and if living in cities. As our study population consisted of mostly highly educated persons, our results could indicate that better-educated persons might be more resilient to environmental challenges. Furthermore, our results might inform sex-specific efforts to prevent becoming overweight at the population level.

## 1. Introduction

The life history theory is a concept that is widely applied in biology. In this concept, risk-taking behaviors are linked with life history traits such as age at first reproduction or birth intervals in many species in predictable ways. Within this framework, “fast” and “slow” life history strategies were defined, where “fast” strategies tend to favor immediate rewards over later advantages, an earlier reproduction, and less attention to the own health. The “slow” strategy defines the opposite [1]. Several studies showed health behavioral adaptations to challenging environments in humans, such as climate change [2].

A line of evidence within this concept focuses on the development of life history strategies during childhood and adolescence, linking life history theory with the developmental origins of adult health [3,4]. In fact, the literature reviews on this topic indicated that children and adolescents growing up in an unpredictable (but not in a stable harsh) environment show a negative impact on life history phenotype developments [5,6]. However, the existence of general life history strategies in humans is still disputed, as it seems that there is little theoretical or empirical evidence in adults that links human behavioral traits with specific life history strategies [7].

The COVID-19 pandemic, with its lockdowns and social restrictions, can be seen as a global increase in environmental uncertainty, potentially leading to negative health implications. In fact, the beginning of the COVID-19 pandemic was characterized by lockdowns and restrictions on everyday life for many populations worldwide. These restrictions had a major impact on people’s behavior and health [8]. The worldwide literature suggested an increase in BMI in children [9,10] and adults [11,12] during the pandemic, aggravating important risk factors for obesity-related diseases [13,14,15]. 

Furthermore, it was shown that the COVID-19 pandemic led to a shift in human decision-making, favoring a preference for immediate gains over future benefits. For instance, an experiment comparing a group of people strongly affected by COVID-19 to a group less affected showed that participants in the risk group tended to ask for more money in order to accept a 30-day waiting period to receive an award than the control group [16]. 

Reports from Europe and Switzerland show differentiated outcomes in different subgroups of the population, with some people increasing healthy lifestyles such as diet and physical activity and others increasing unhealthy behaviors. It seems that factors such as age, sex, urbanization, and socioeconomic position modulated the impact of the COVID-19 restrictions on health outcomes [17,18,19,20,21]. This phenomenon can be discussed within the life history theory where an unpredictable environment should lead to an “unhealthier” lifestyle, especially in socioeconomically underprivileged people [22,23].

A better understanding of these behavioral changes and the underlying factors is essential to better understand the overall rules of human reaction norms, especially as a consequence of local or global disasters. Such knowledge can lead to an increase in populations’ resilience to such events and to more efficient preventive strategies to mitigate negative health outcomes. The present study aims to investigate factors influencing diet, physical activity, and BMI during and after the end of the COVID-19 restrictions in Switzerland in an ongoing cohort study. Our results shall contribute to the development of more personalized and better risk-group targeted overweight preventive strategies, especially if following major environmental challenges.

## 2. Materials and Methods

### 2.1. Study Design and Participants

The data presented in this paper derive from an ongoing cohort study that started in 2019 at the Institute of Evolutionary Medicine at the University of Zurich, in collaboration with ETH Zurich. Participants from the Swiss Food Panel Study 2.0 from the ETH Zurich [24,25] were invited in 2019 to participate in a cohort study, where apart from diet and physical activity, anthropometry (stadiometer, balance, 3D body scanner) and body composition (bioimpedance analysis, BIA) were measured in annual intervals. The inclusion criteria were the adult general population, able to complete a questionnaire in German, both sexes, and all ages over 18 years. The exclusion criteria were pregnancy, implanted electronic devices, and severe consuming diseases, as these conditions alter body composition and electronic devices might be influenced by BIA. In 2020, there were no measurements due to the COVID-19 pandemic. As only part of the 2019 cohort returned after the pandemic (130 out of 240), from 2021 onward, the cohort was expanded by email invitation of people working at the University of Zurich. In the present paper, only participants who started in 2019 and have at least one follow-up measurement 2021–2023 are included. All study participants were informed orally and in writing about the study procedure. Informed consent was obtained from all subjects involved in the study. 

### 2.2. Questionnaire

The study participants completed a questionnaire on their average dietary intake of several food groups during the past year. The questionnaire was designed to allow the calculation of a Diet Quality Index [26], which is based on the Swiss national dietary recommendations [27]. This questionnaire was adapted from a previously published version by ETH [24,26] and was based on the validated food frequency questionnaire of the Nurses’ Health Study [28]. The questionnaire also asked questions about physical activity at work and during leisure time [29], as well as sociodemographic variables such as sex, age, place of residence, and education level.

### 2.3. Outcomes and Variables

The main outcome body mass index (BMI) was calculated from measured weight and height as kg/m^2^. The participants’ height was measured with a standard stadiometer (Seca 274). Weight was measured with a balance incorporated in the segmental medical 8-point body composition analyzer (BIA) (Seca mBCA 515, Seca AG, Reinach, Switzerland) [30]. As BMI was not normally distributed, we applied a natural logarithm transformation to the BMI values for statistical analyses.

Using five food categories of the food frequency questionnaire, we calculated a diet quality index [26]: fruits, vegetables, whole grain products, meat, and sweet/salty snacks. Following the official Swiss diet recommendations, we divided all food intake amounts in each food category into two groups, depending if recommendations were met or not. For each participant and each of the five categories, one point was assigned if the diet recommendation was met or zero points if the recommendation was not met. In this way, a score from 0 to 5 could be formed, which correlated with the overall healthiness of the individual diet [26]. For sample size reasons, the score was further summarized into three categories: unhealthy diet quality (0–1 points), medium diet quality (2–3 points), and healthy diet quality (4–5 points). 

Physical activity during leisure time was assessed using a published questionnaire and giving examples [29]. Categories included 1. very light (almost no physical activity), 2. light (e.g., walking, slow biking), 3. moderate (e.g., running, biking), 4. heavy (e.g., intensive running, intensive biking), and 5. very heavy (exhaustive activity several times per week). For sample size reasons, data were summarized into three categories: light physical activity (categories 1–2), moderate physical activity (category 3), and heavy physical activity (categories 4–5). 

Education was assessed using Swiss educational categories: 1. mandatory education, 2. basic education, 3. professional training, 4. high school, 5. higher professional studies, 6. higher education, and 7. university. For sample size reasons, the data were summarized into primary/secondary education (categories 1–4) and tertiary education (categories 5–7). 

Age was calculated from the year of birth for each of the measurement years and categorized into four quartiles, as age was not normally distributed. The place of residence was categorized as 1. urban, 2. suburban, and 3. rural. 

### 2.4. Statistics

Data were analyzed graphically, descriptively, and analytically. We used *t*-tests to compare continuous variables and chi-squared tests to compare categorical variables between the sexes for the baseline years 2019 and 2021. To assess changes in categorical variables between the study years, we used repeated-measures ANOVA, separated by sex. We used multilevel mixed-effects linear regression models to assess changes in BMI over the measurement years, separately for each sex, and corrected for diet, physical activity, age, education, and place of residence. This model allows us to assess repeated measurements of individuals, considering fixed effects as well as random effects of the study year. 

## 3. Results

### Descriptive Results

**Figure 1:**
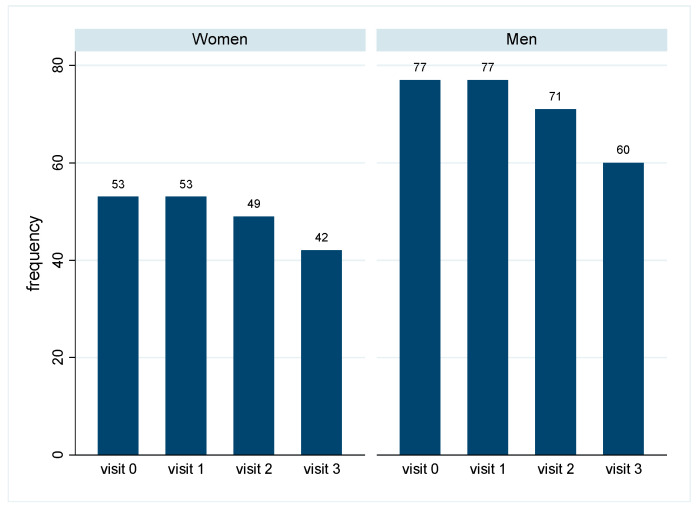
Number of participating women and men in the years 2019–2023: Number of participants for each study year, separately by sex. Visit 0: 2019 (baseline), visit 1: 2021, visit 2: 2022, visit 3: 2023.

In total, 53 women and 77 men started the cohort study in 2019 and showed at least one follow-up measurement in the post pandemic years 2021–2023. Some participants dropped out over time, and in 2023, 42 women and 66 men were still participating in the study (Figure 1). The reasons for dropping out were known in a few cases only and included explanations such as advanced age, not wanting to travel, not wanting to spend money, or travel abroad.

**Figure 2:**
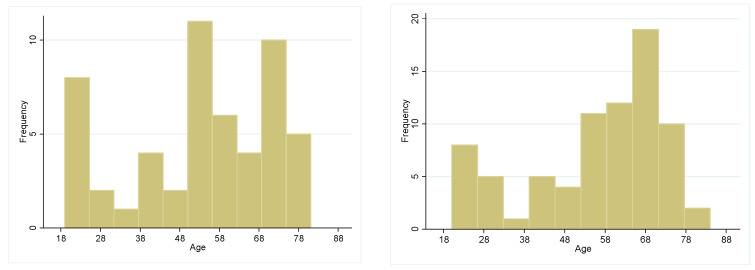
Age frequencies distribution in men and women at baseline.

At the baseline in 2019, the mean age in women was 53.43 years (SD 18.5) and in men it was 56.03 years (SD 17.1) (*p* > 0.05). For age distribution in both sexes, see Figure 2. Women had a lower BMI (mean 23.05, SD 3.05) than men (mean 25.11, SD 3.21) (*p* < 0.001). Most women reported a medium-quality diet while most men showed a low-quality diet (*p* < 0.01). This sex difference persisted after the pandemic in 2021 (*p* < 0.01).

Women reported a medium-level physical activity in 2019, while men reported a high-level physical activity (*p* = 0.001). Again, this sex difference persisted after the pandemic in 2021 (*p* < 0.01). In both sexes, most participants showed a tertiary education (65.3% in women, 72.4% in men, *p* < 0.05). While most women lived in agglomerations, most men lived in cities (*p* > 0.05). For detailed results, see Table 1.

**Figure 3:**
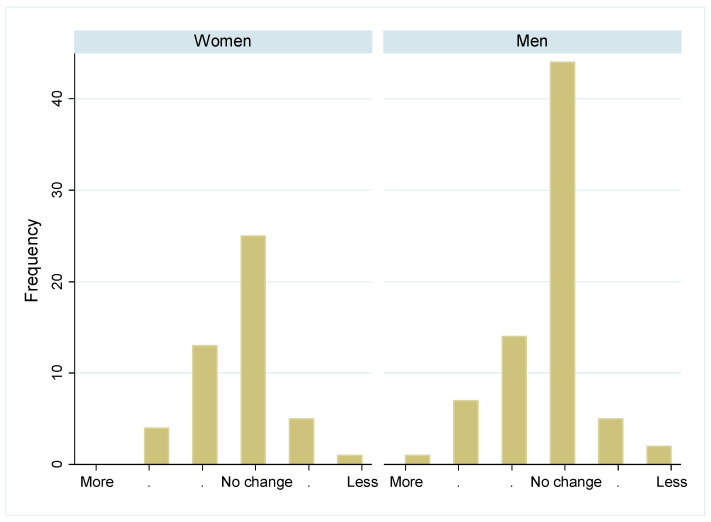
Change in daily food quantity intake in women and men during the pandemic: Number of participants who changed their daily food quantity intake during the pandemic. Categories include “much more”, “more”, “slightly more”, “no change”, “slightly less”, “less”, and “much less”. Numbers are given separately by sex.

**Figure 4:**
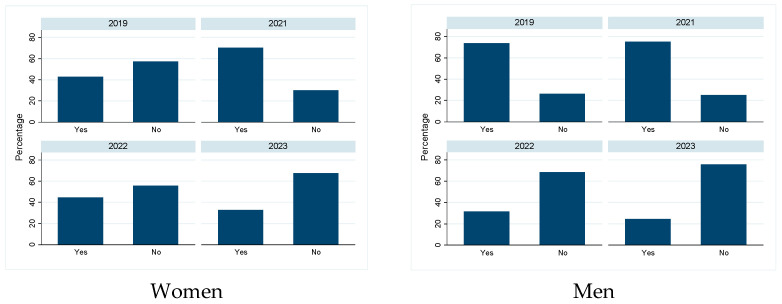
Percentage of participants following a weight loss diet: Number of persons per study year who followed a weight loss diet (yes) or not (no), separated by sex.

**Figure 5:**
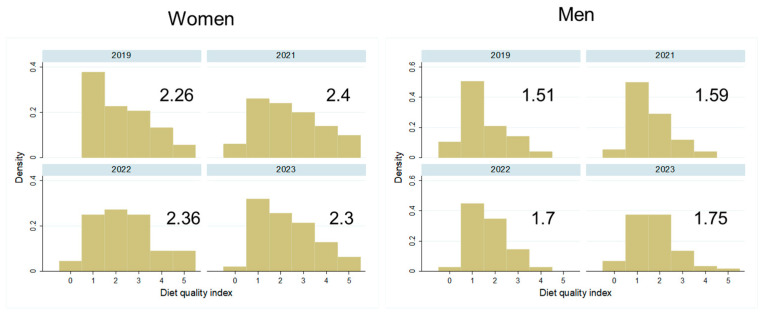
Distribution and mean diet quality index in women and men over the years 2019–2023: Distribution of participants in the five categories of diet quality, from low to high quality, by study year and separately by sex. The mean value for diet quality is given for each study year.

**Figure 6:**
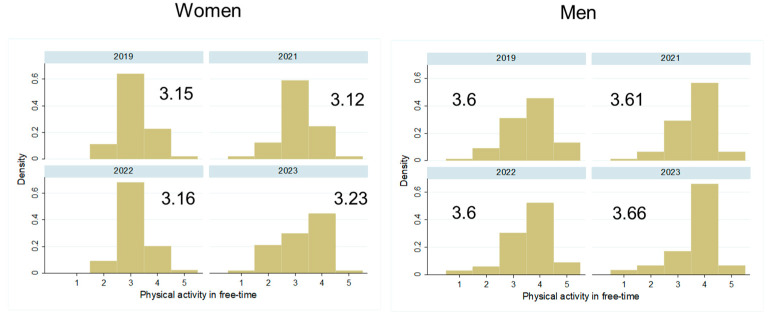
Distribution and mean physical activity level in women and men over the years 2019–2023: Distribution of participants in the five categories of physical activity, from low to high intensity, by study year and separately by sex. The mean value for physical activity is given for each study year.

Most participants reported not to have changed the quantity of their food intake during the pandemic, and there was no difference between the sexes in this self-evaluation (Figure 3, *p* > 0.05). While some more women followed a weight-loss diet shortly after the pandemic (Figure 4 left, *p* > 0.05), significantly fewer men followed a weight-loss diet since 2022 (Figure 4 right, *p* < 0.001). Diet quality did not change significantly between 2019 and the post pandemic years, in both sexes (Figure 5, *p* > 0.05). Similarly, the physical activity level did not change significantly over time in both sexes (Figure 6, *p* > 0.05).

**Figure 7:**
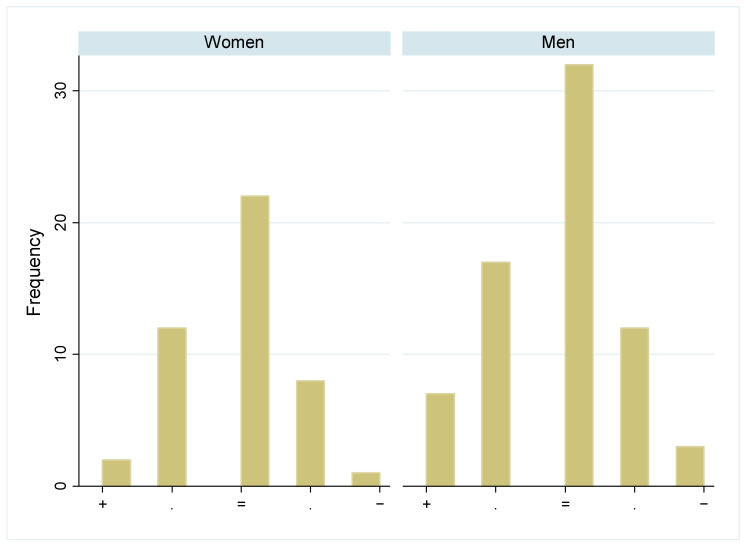
Change in perceived weight in women and men during the pandemic: Number of participants who were believed to have changed their weight during the pandemic, in five categories from weight gain (+) to weight loss (−), separated by sex.

**Figure 8:**
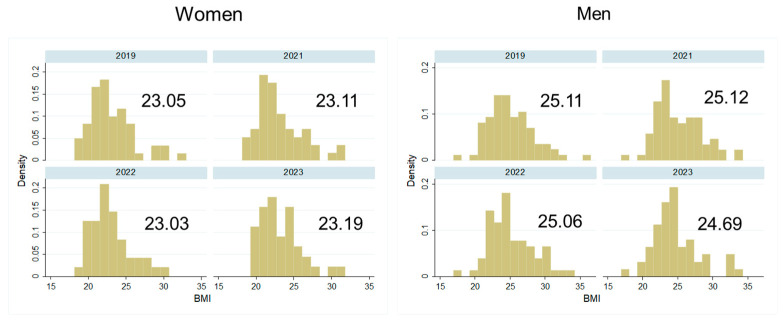
Measured BMI distribution and mean BMI values in women and men over the years 2019–2023.

Most participants in both sexes reported no significant changes in body weight during the pandemic (Figure 7, *p* > 0.05 for sex difference). Indeed, if measured BMIs were compared over the years 2019–2023, there was no significant change over time in either sex (Figure 8, *p* > 0.05). In the analytical models, in women, physical activity and age were associated with BMI (all *p* < 0.05), while in men, apart from physical activity, diet quality and living place were associated with BMI. While high physical activity levels and a high diet quality (for men) were associated with a lower BMI, higher age and living in the agglomeration (for men) were associated with a higher BMI.

## 4. Discussion

In this study, we assessed whether there were changes in BMI as well as in eating and physical activity patterns during and shortly after the COVID-19 pandemic in a general population in Switzerland. The worldwide literature suggested an increase in BMI in children [9,10] and in adults [11,12] during the pandemic, aggravating important risk factors for obesity-related diseases [13,14,15]. Interestingly, we did not find any significant changes in BMI or lifestyle factors over this time in our study sample, except for less dieting behavior in men. One reason might be the small sample size of our study. However, a constant BMI during and after the pandemic was also found in an analysis of Swiss conscription data, where over 370,000 young men in Switzerland were included [31]. In a cohort of Swiss children, an increase in BMI during the pandemic was recorded for obese children, but much less so for normal-weight children [32]. 

A population-wide survey on the impact of the COVID-19 lockdown in Switzerland showed that most people did not change their eating or physical activity habits much, with 7% of people moving more than before, and 22% less. These changes rapidly disappeared again after the lockdown [17]. Another Swiss survey showed that this was especially true for people under the age of 45 years who changed their lifestyle habits during the pandemic, while elderly people did so much less. Some people increased their intake of healthy food, while others increased their intake of unhealthy food [18]. Similarly, a study on adults showed that people working in home-offices during the 2020 lockdown in Switzerland reacted partially with an increase in healthy food choices and partially with a decrease [19]. People living in cities and showing a lower socioeconomic level changed more often toward an unhealthy lifestyle than people living in rural areas of Switzerland [18]. Similar results were found in Italian adults, showing different reaction types among humans exposed to the COVID-19 lockdown situation [20]. Likewise, a study in Australia reported a partial increase and a partial decrease in physical activity during the COVID-19 pandemic [21]. In summary, it seems that people of higher socioeconomic status, living in cities, who were normal weight and showed a healthy lifestyle before the pandemic tended to keep their weight, increase the healthiness of their diet, and increase their level of physical activity during the pandemic, while people of lower socioeconomic status, living in rural areas, with overweight and unhealthy lifestyle habits tended to deteriorate. As our study consisted of mostly highly educated and elderly people living in cities or city agglomerations, our results align with previous findings. These findings are also in line with life history strategies that predict a stronger reaction toward unhealthy behaviors in younger people and in people with a lower socio-economic status [22,23].

Several studies outside of the realm of the COVID-19 pandemic showed that humans adapt their health behavior (e.g., diet, physical activity) according to changes in the environment, especially when facing harsh environmental conditions during childhood [2,5]. 

In Switzerland, only a few persons experienced harsh or unpredictable environmental conditions in childhood (if immigrants are excluded). The absence of major wars or natural disasters since the mid-20th century in large parts of Europe led to what was described in the literature as the “disaster gap”. This term describes the loss of functional disaster memory after a long period with few or no disasters in a country or region [33]. This effect was especially pronounced in affluent post-WWII Switzerland [34] and could additionally contribute to the fact that our highly educated Swiss study population did not react with an “unhealthier” life history strategy during the COVID-19 pandemic.

Another interesting aspect of our study concerns physiological and behavioral sex differences. In Europe, approximately 60% of the population is overweight or obese, with men showing higher levels than women [35]. In Switzerland, overweight levels are generally lower than in Europe [36]. We can confirm sex differences in BMI in our study sample with women showing lower levels, before and after the pandemic. Concerning lifestyle behaviors, women reported higher diet quality, but less physical activity than men, before and after the pandemic. After the correction for sociodemographic factors, in women, physical activity and age were associated with BMI, while in men, apart from physical activity, diet quality and place of residence were associated with BMI. While high physical activity levels and a high diet quality were associated with a lower BMI, higher age and living in a city agglomeration were associated with a higher BMI. In women, the role of age for BMI could be linked to menopausal status [37]. Apart from the general effect of an increase in BMI with increasing age, our sample, which includes many post-menopausal women, could also show an effect of hormonal changes impacting female body composition. Interestingly, a systematic review of weight management studies did not show clear sex differences concerning weight influencing factors. However, this study noted a widespread underreporting of sex-specific results in weight loss studies [38]. 

From a life history perspective, a sex difference in health behaviors can be expected, with men tending to an “unhealthier” life strategy (e.g., higher risk-taking, more aggression), while women generally show a “healthier” life strategy [39,40]. Our finding that men were more physically active but had unhealthier diets compared to women aligns with this perspective. The role of place of residence for BMI in men is probably more linked to education levels, as in Switzerland, tertiary-educated people more often live in cities. Our hypothesis is that lower-educated men more often live in agglomerations and at the same time more often follow an “unhealthy” life strategy and consequently show a higher BMI. These associations should be confirmed in a larger population with different levels of education. If this hypothesis holds true, this would have an impact on better risk-group-targeted overweight preventive strategies. 

This study shows strengths and weaknesses. Strengths include the comprehensive behavioral questionnaire and the measurement of anthropometry with medical devices. Many studies rely on body weight and height data sampled with questionnaires, but self-reported data are prone to recall bias and social desirability bias, and studies showed that women tend to underreport their weight, while men tend to overreport their height [41]. A weakness of the present study is the small sample size and the sample composition, which is not representative of the Swiss general population. In fact, our study sample consists of mostly highly educated persons with a healthier BMI and lifestyle than the Swiss general population [36,42]. To generalize our findings, more research in a larger and more representative sample is needed.

## 5. Conclusions

Our study did not show major changes in BMI or health behavior during or after the COVID-19 pandemic in a sample of highly educated adults in Switzerland. Our results could indicate a higher resilience in highly educated persons toward environmental challenges, which confirms predictions from the life history theory. A better understanding of modulating factors such as sociodemographic or behavioral differences is essential to better understand the overall rules of human reaction norms, especially following local or global disasters. Such knowledge can lead to an increase in the population’s resilience to such events, and to more efficient preventive strategies to mitigate negative health outcomes.

Furthermore, we noted sex differences in anthropometry and health behaviors that are in line with life history strategies. As many studies do not report their results separately by sex, a comparison between studies is difficult. We therefore encourage the reporting of sex-specific results in studies on human physiology and behavior. Our results shall contribute to the development of more personalized and better risk-group-targeted overweight preventive strategies.

## Figures and Tables

**Table 1:** Baseline descriptive data of the study participants in 2019.

Variable	Women	Men
Sample size (n)	53	77
Mean age in years (SD)	58.4 (18.5)	61.0 (17.1)
Mean BMI (SD)	23.05 (3.05)	25.11 (3.21)
DQI unhealthy (%)	37.7	61.0
DQI medium (%)	43.4	35.1
DQI healthy (%)	18.9	3.9
Physical activity level low (%)	11.3	10.4
Physical activity level medium (%)	64.2	31.2
Physical activity level high (%)	24.5	58.4
Non-tertiary education (%)	34.7	27.6
Tertiary education (%)	65.3	72.4
Residence city (%)	42.2	40.0
Residence agglomeration (%)	48.9	33.3
Residence rural (%)	8.9	26.7

DQI = Diet quality index.

## Data Availability

The datasets presented in this article are not readily available because the data are part of an ongoing study. Requests to access the datasets should be directed to N.B.
